# Demographic characteristics and anxiety in the educational setting during the COVID‐19 pandemic in Indonesia: A cross‐sectional study

**DOI:** 10.1002/hsr2.792

**Published:** 2022-08-18

**Authors:** N. Juni Triastuti, Erna Herawati

**Affiliations:** ^1^ Department of Medical Education Universitas Muhammadiyah Surakarta Indonesia; ^2^ Department of Psychiatry Universitas Muhammadiyah Surakarta Indonesia

**Keywords:** age, anxiety, COVID‐19, demographic, educational

## Abstract

**Background and Aims:**

This study aims to analyze the relationship between gender, age, occupation, residence, and anxiety in the education environment during the coronavirus disease 2019 (COVID‐19) pandemic.

**Methods:**

This study used a descriptive‐analytic cross‐sectional design to determine anxiety using the DASS 42 questionnaire given to 181 respondents. The sampling technique used was purposive sampling, and the data analysis used was the Chi‐square test and multivariate analysis.

**Results:**

It was found that 66.7% of teenagers experienced anxiety, while 33.3% of adults experienced anxiety. In addition, the school‐age community (86.2%) experienced higher anxiety compared with the working‐age community (13.8%) who experienced anxiety. Women experienced more significant anxiety (66.7%) compared with men (33.3%). People living on the island of Java (74.7%) have a greater incidence of anxiety compared with people living outside Java (25.3%).

**Conclusion:**

There is a significant relationship between the type of occupation and the incidence of anxiety with *p* < 0.05 (OR = 0.341). A significant correlation was found between age with the incidence of anxiety with *p* < 0.05 (OR = 0.489). The demographic factors altogether had significant relationships with the anxiety in the educational environment during the COVID‐19 pandemic with a *p* value < 0.05 and R Square of 0.069. There is a strong relationship between the demographic factors and the incidence of anxiety in the community's educational environment during the COVID‐19 pandemic with *p* < 0.05. It is suggested that women, school‐age communities, or the un‐employment community need to be supported to alleviate the impact of COVID‐19 on anxiety through several programs.

## 1 INTRODUCTION

Society around the globe is currently facing a coronavirus disease 2019 (COVID‐19) pandemic. This pandemic has occurred from the end of 2019 until now.^1^ The various impacts that have arisen due to this pandemic include public anxiety regarding the relatively high incidence of COVID‐19.^2^ Meanwhile, little is known about the prevalence of anxiety in the educational setting during the COVID‐19 pandemic.

Anxiety or usually recognized as Generalized Anxiety Disorder (GAD) is defined as a feeling of insecurity, fearfulness, tension, and fear that arises as a result of an unpleasant event and persists for at least 6 months or more.^3^ According to Henriksson et al.,^4^ anxiety is an emotional condition that is an unpleasant feeling, involving psychophysiological responses that occur as a result of anticipating the risk that threatens, and exercise is suggested to provide healing to the anxiety and depression.

The estimated prevalence of anxiety disorders in the United States is more than 23 million people each year, which is about 19.1% of individuals who experience an anxiety disorder.^5^ Anxiety is a mental disorder whose age and lifetime prevalence increased from August to December 2020 during the COVID‐19 pandemic.^6^ It is reported that women are more prone to anxiety disorders than men.^1^, ^7^, ^8^ Anxiety disorders occur in about 29.7% compared with 19.3% of mood disorders.^7^ Meanwhile, during the COVID‐19 pandemic the prevalence of anxiety increased by around 25%.^9^, ^10^ During the pandemic, the number of populations who took the prescription and counseling treatment for the mental health increased to around 25% from previously 22.4% during late August until early December 2020.^11^

The research reported in the first month of the COVID pandemic indicated that the anxiety cases in Indonesia were reported at around 20%.^8^

Several factors contribute to the incidence of the anxiety during COVID‐19 pandemic which encompasses gender; COVID‐19 patients; lack of social assistance.^8^ Providing sufficient information on the COVID‐19, accessible healthcare system (supportive relationship), and social assistance are believed to mitigate the impact of COVID‐19 on the anxiety.^8^

The mechanism of the anxiety in the COVID‐19 pandemic is related to the inflammation marker, following the oxygen saturation level exacerbated in the patient with COVID‐19.^12^

Several theories explain the factors for the occurrence of anxiety disorders,^5^, ^13^ namely: (1) Psychoanalytic theory which this theory states that anxiety occurs due to emotional conflict between two psychological structures, specifically the id and the super‐ego. (2) Behavioral theory explains that anxiety occurs as a result of an excessive response to a specific or learned stimulus from an individual experience. (3) Existential theory, where there is no specific stimulus for chronic anxiety disorders. (4) Biological theory, this theory shows the three main neurotransmitters associated with anxiety disorders including norepinephrine, serotonin, and gamma‐aminobutyric acid (GABA).^14^

Some of the triggering factors are sourced from the external environment such as the entry of bacterial infections, viruses, a place to live that is not conducive to certain traumas, disorders of self‐concept such as divorce from parents, loss of someone, loss of work, changes in social culture and education, anxiety among parents.^14^ Meanwhile, internal factors can also affect anxiety such as a lack of functioning of the immune system, cardiovascular disease disorders, interpersonal conflict disorders, and changes in relationships and roles.^14^

Norepinephrine plays an important role in the mechanism of anxiety disorders, caused by anxiety disorders, poor noradrenergic limbic regulation, or excessive activity.^15^ Serotonin is a monoamine neurotransmitter that acts at the synapses of nerve cells. Many serotonin receptors have an important role in the pathogenesis of anxiety, namely the 5‐HT1A receptor which has the widest distribution of other receptors. The presence of serotonergic is the cause of anxiety.^16^

Gamma‐aminobutyric acid or GABA is the most widespread inhibitory neurotransmitter in the central nervous system. The presence of increased GABA activity and dysregulation or poor GABA‐anergic regulatory centers causes anxiety. The role of GABA in the mechanism of anxiety is supported by the benefits of benzodiazepines that increase GABA activity against GABA‐A in the treatment of several anxiety disorders.^17^

Symptoms that are usually complained of by people with anxiety disorders include Anxious, bad feelings, worry, anxiety, afraid of his mind, easily offended, and hopelessness. The other symptoms encompass feeling unsettled, tense, restless, easily startled, afraid of crowds, and afraid of being alone. Sleep disturbances, nightmares, impaired concentration, and memory can also be experienced by the patients. Somatic symptoms such as muscle and bone pain, palpitations, shortness of breath, headache, etc., gastrointestinal disorders, ringing in hearing (tinnitus), and urogenital disorders.^11^, ^18^

This study needs to be done to assess the incidence of anxiety that occurs in the primary educational environment therefore factors can be identified that can be a risk for anxiety events during the COVID‐19 pandemic. The research data regarding the incidence of anxiety during the COVID‐19 pandemic that discusses events in the educational environment are still absent, particularly in Indonesia. The study related the anxiety to the COVID‐19 pandemic is limited in Bangladesh that conveyed data on the number of people experiencing anxiety and only focused on undergraduate students (Islam M.A, 2020) and data that expressed the incidence of anxiety in the educational environment in Indonesia is still very limited. One study explored the anxiety in the initial phase of COVID‐19 with the respondents who were not focusing on the educational setting.^8^ Hence, this study extends the knowledge using different respondents both from the educational staff or teachers and the students from different levels of education from high school to undergraduate students, and from different ages of 17‐64 years.

The research question of this study is whether there are relationships between the factors of age, gender, place of residence, and type of work on the incidence of anxiety in the Indonesian education community.

## 2 MATERIALS AND METHODS

**Figure 1:**
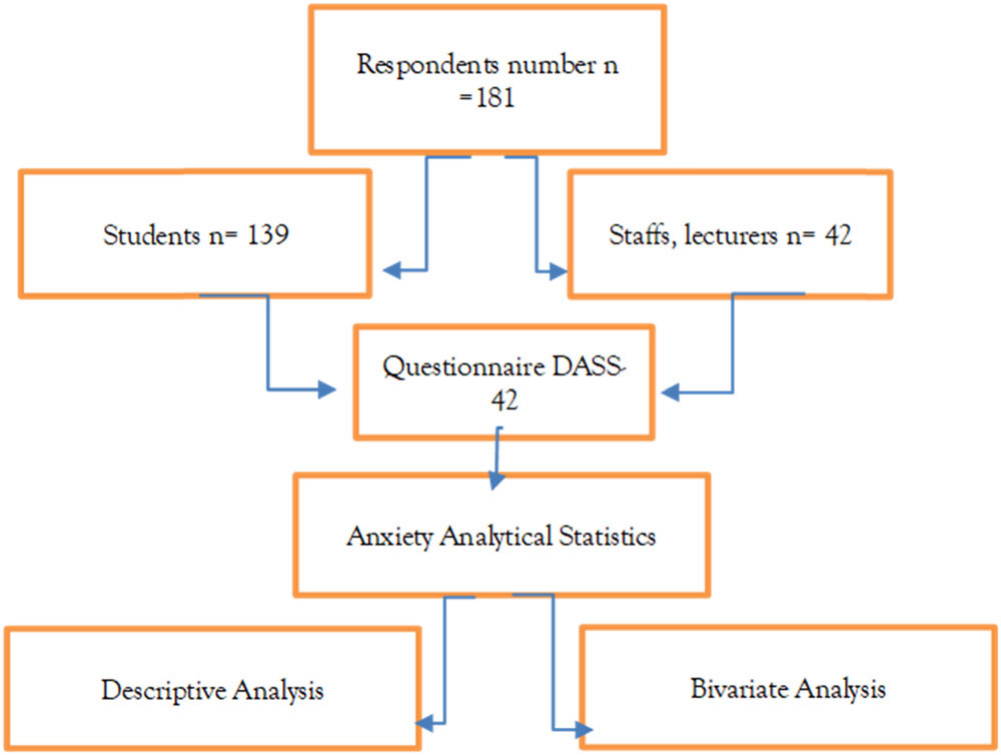
The research method flow chart. DASS, Depression Anxiety and Stress Scale

To answer the research questions, the researcher used an analytical observational method using the Chi‐Square test and multivariate analysis to analyze the data. The method of data collection is illustrated in Figure 1.

### 2.1 Study design and participants

This study used an observational analytic research design with a cross‐sectional study approach, in which the independent and dependent variables are assessed at the same time.

Samples were taken with the following inclusion criteria: People who live in the environment in Indonesia and or Indonesian citizens 14–75 years old, who were willing to become research respondents, and who are involved in the educational environment, both as students, staff and teachers/lecturers. Exclusion Criteria included residents who cancel to take part in the study, and who did not fill out the questionnaire. This study used a purposive sampling technique, that selected samples based on certain criteria.^19^, ^20^, ^21^ The minimum sample size in this study was determined using the rule of thumb formula based on the number of independent variables. One of the guidelines in the rule of thumb is that the number of samples required is between 5 and 50 times the number of independent variables studied. To narrow the confidence interval of the research results, in this study a multiplication factor of 35 times the number of independent variables was determined.^19^, ^20^, ^21^ In this study, there were four independent variables studied thus a sample size of 35X the number of independent variables was determined and was obtained in 140 samples. To avoid samples that drop out, the number of samples is added by 10% thus the required number of samples was 154 samples. The number of respondents who met the inclusion criteria was 181 samples, therefore the number of samples of 181 had exceeded the minimum number of samples required (154 samples).^19^ Respondents were then asked to fill out an online questionnaire.

### 2.2 Data collection

The location of data collection in this study was carried out in the environment and community in the territory of Indonesia. The data collection was from March–May 2021.

The questionnaire in the online form (Google Form) was sent to the prospective respondents through online platforms such as WhatsApp and Instagram and to be circulated to friends or colleagues. Upon filling out the questionnaire, the respondents have agreed to consent to participate in the study by filling out the questionnaire.

All the prospective respondents which have occupations either as teachers/lecturers/educational staff, or students (high school or undergraduate) can fill the questionnaire if they wish.

### 2.3 Measures

The survey in this study used Depression Anxiety and Stress Scale (DASS) Questionnaire which was first developed by Lovibond and Lovibond^22^ and then adapted in Indonesia by Damanik and Rusli.^23^

The DASS questionnaire consists of 42 items that measure general psychological distress. The anxiety scales consist of 14 statement items. The value obtained from the respondent's response is categorized according to the level of the respondent's psychological disorder. Anxiety level responses were categorized into 2, namely scores with a score of not being anxious ≤ 7 and experiencing anxiety with a score of >7.^24^, ^25^ The demographics information which was presented in this study included age, gender, employment, and place of residence.

The reliability test used Cronbach's alpha, it was found that Cronbach's alpha value was 0.963, which indicates that all questions on the DASS questionnaire have very high (perfect) reliability because the value >0.9.

### 2.4 Statistical/data analysis

The research data was analyzed using SPSS Ver 26. computer software. Data analysis comprised descriptive univariate, bivariate, and multivariate analysis. To explain the relationship between the dependent variable and the independent variable, the statistical test used is the Chi‐Square test (*χ* ^2^). The chi‐square test is used in this study because the chi‐square test is a data analysis technique that can be used to test hypotheses if there are two or more classes in the population where the independent variable data is nominal and the sample is large.^(16)^ In this study, the analyzed data has met the requirements of using Chi‐square, which indicated there were no cells with a value of zero and the number of cells with an expected count of less than 5 was not more than 20%.^21^, ^26^ The analysis employed a 95% confidence Interval (CI) for the level of significance using two‐side The analysis was then continued to multivariate analysis. The regression logistic was applied to test whether all independent variables as predicting factors are associated with anxiety.^27^, ^28^ In reporting the analysis, this study followed the SAMPL guidelines and STROBE guidelines for cross‐sectional studies reports (https://www.equator-network.org). This study has been granted the Ethics Approval from the Faculty of Medicine Universitas Muhammadiyah Surakarta. (Reference number. 3390/B.2/KEPK‐FKUMS/III/2021).

## 3 RESULT

Based on the research data obtained from the DASS Questionnaire through the online platform (Google form), revealed that 181 respondents agreed to fill out the questionnaire. The outliers of the data were minimal as indicated in the mean and median values which were quite close (Table 1). In this study, the characteristics of the respondents were divided by age, gender, place of residence, and the respondent's level of anxiety (Table 1).

**Table 1:** Subject characteristics and bivariate analysis (*n* = 181)

Characteristics	Total	Percentage	Not experiencing anxiety	Experiencing anxiety	Mean (SDs)	Median (range)	Chi‐Square) *p* value	Odd Ratio (95% CI)	Logistic regression (*p* value)	R square
Gender									<0.001	0.069
Male	71	39.2	42 (44.7%)	29 (33.3%)	1.39 (±0.49)	1.00 (1.00)	<0.5	0.619 (0.339–1.132)		
Female	110	60.8	52 (55.3%)	58 (66.7%)						
Age										
17‐23 years old	104	57.8	46 (49.5%)	58 (66.7%)	2.58 (±0.72)	1.00 (1.00)	<0.05	0.489 (0.262–0.875)		
24‐64 years old	76	42.2	47 (50.5%)	29 (33.3%)						
Residential										
Java Island	140	77.3	75 (79.8%)	65 (74.7%)	1.387 (±0.81)	1.00 (1.00)	<0.5	1.336 (0.665–2.685)		
Outside Java Island	41	22.7	19 (20.2%)	19.7 (25.3%)						
Employment										
Working	42	23.2	30 (31.9%)	12 (13.8%)	1,23 (±0.42)	1.00 (1.00)	<0.05	0.341 (0.162–0.721)		
Not working (School)	139	76.8	64 (68.1%)	75 (86.2%)						

Abbreviations: CI, confidence interval; SDs, standart deviations.

Respondents are categorized as not having anxiety if the anxiety level value is 7, while the total anxiety value is >7 then categorized as experiencing anxiety.^24^, ^25^

Based on Table 1 above, it was found that as many as 60.8% (*n* = 110) were female while the male respondents were 39.2% (*n* = 71). Women experienced more anxiety as much as 58 66.7% (*n* = 58) compared with men who suffered from anxiety as much as 33.3% (*n* = 29). However, the significance value which shows the relationship between gender and the incidence of anxiety is less significant, which is indicated by a *p*‐value <0.5 with an OR value of 0.619.

In this study, the age category grouped into 17–23 years was 57.8% (*n* = 104) more than the 24–64 years age group, which was 42.2% (*n* = 76). In this age group, it was found that adolescents aged 17–23 years were found to experience more anxiety, namely 66.7% (*n* = 58) when compared to adults aged 24–64 years, which were 33.3% (*n* = 29). Data analysis shows that the age factor has a strong relationship with the incidence of anxiety as evidenced by a strong significance value with a *p*‐value of <0.005 with an OR value of 0.489. This shows that the factor of adolescent age or school age is more prone to experiencing anxiety events than adults or not in school‐age where adolescents tend to experience anxiety which is 0.489 times higher than adults.

**Figure 2:**
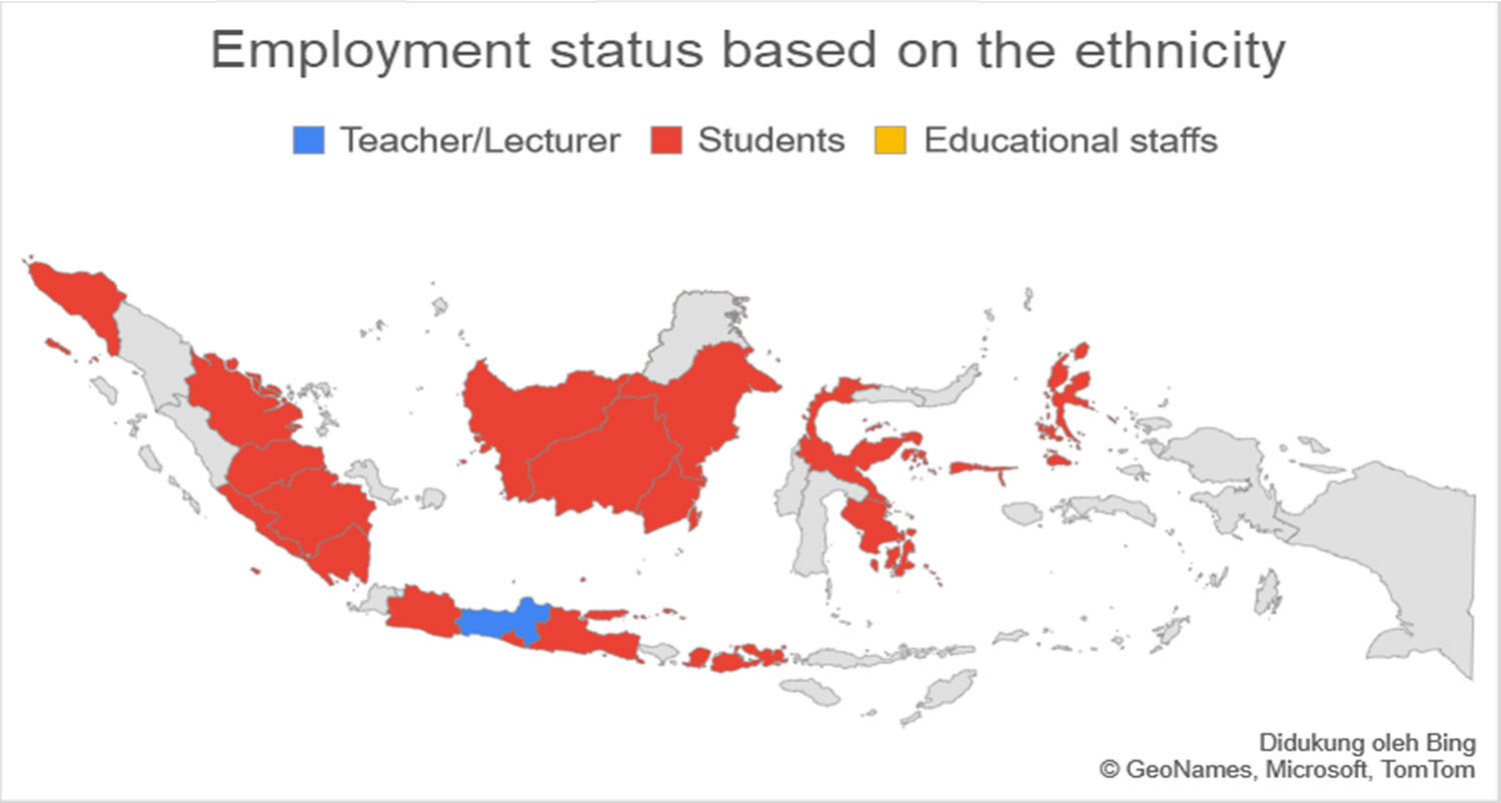
Employment status based on the ethnicity (primary source).

**Figure 3:**
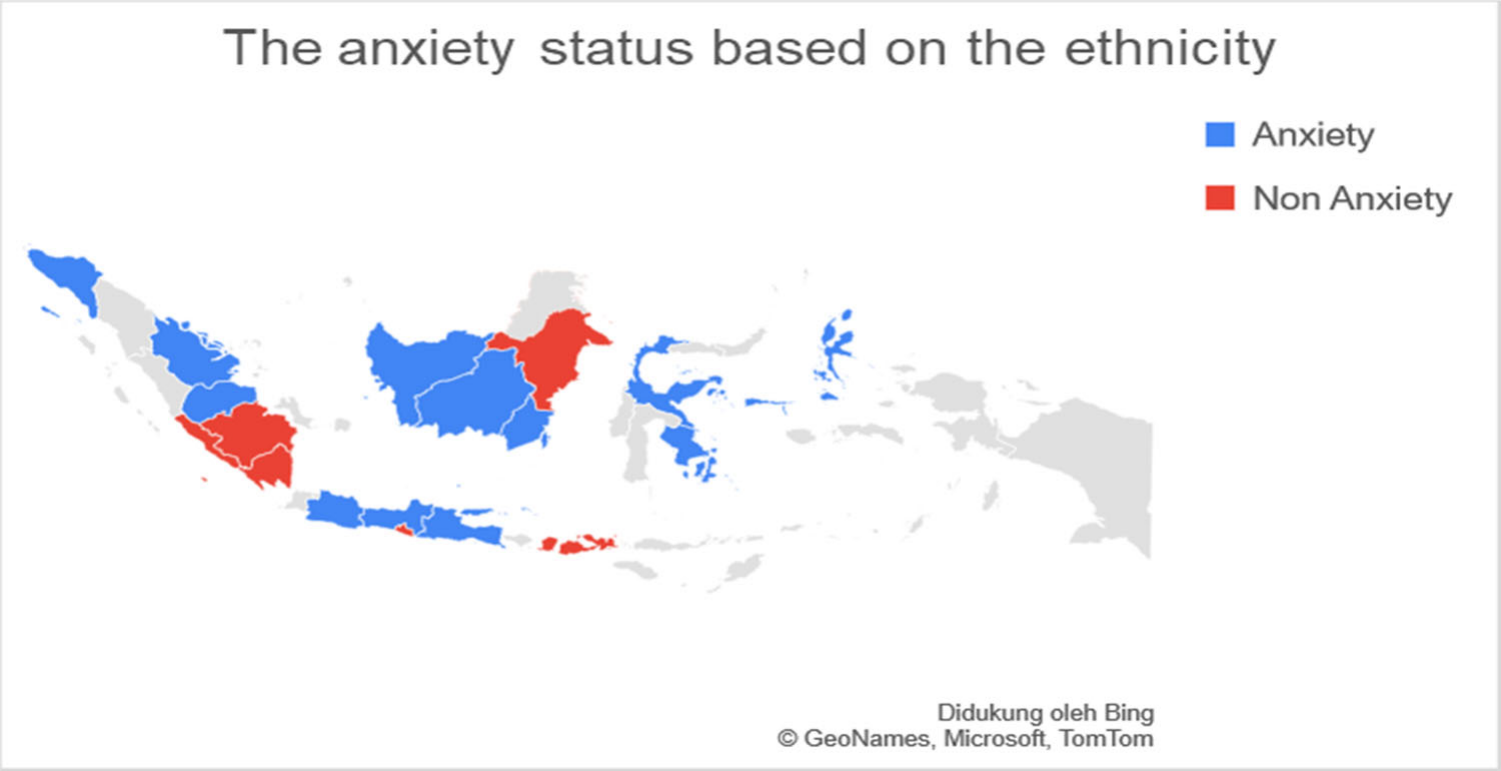
The anxiety status is based on the ethnicity (primary source).

**Figure 4:**
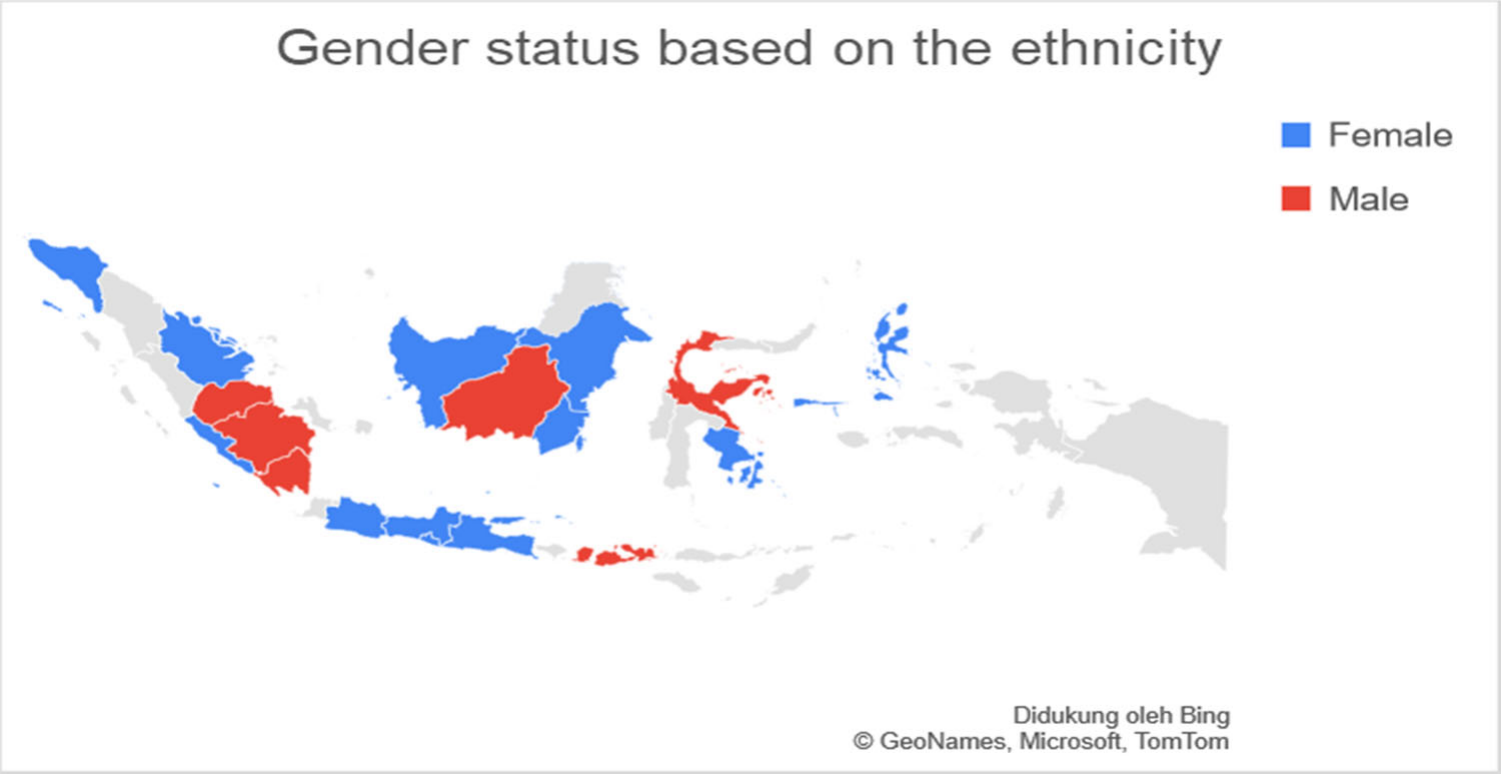
The gender status is based on the ethnicity (primary source).

**Figure 5:**
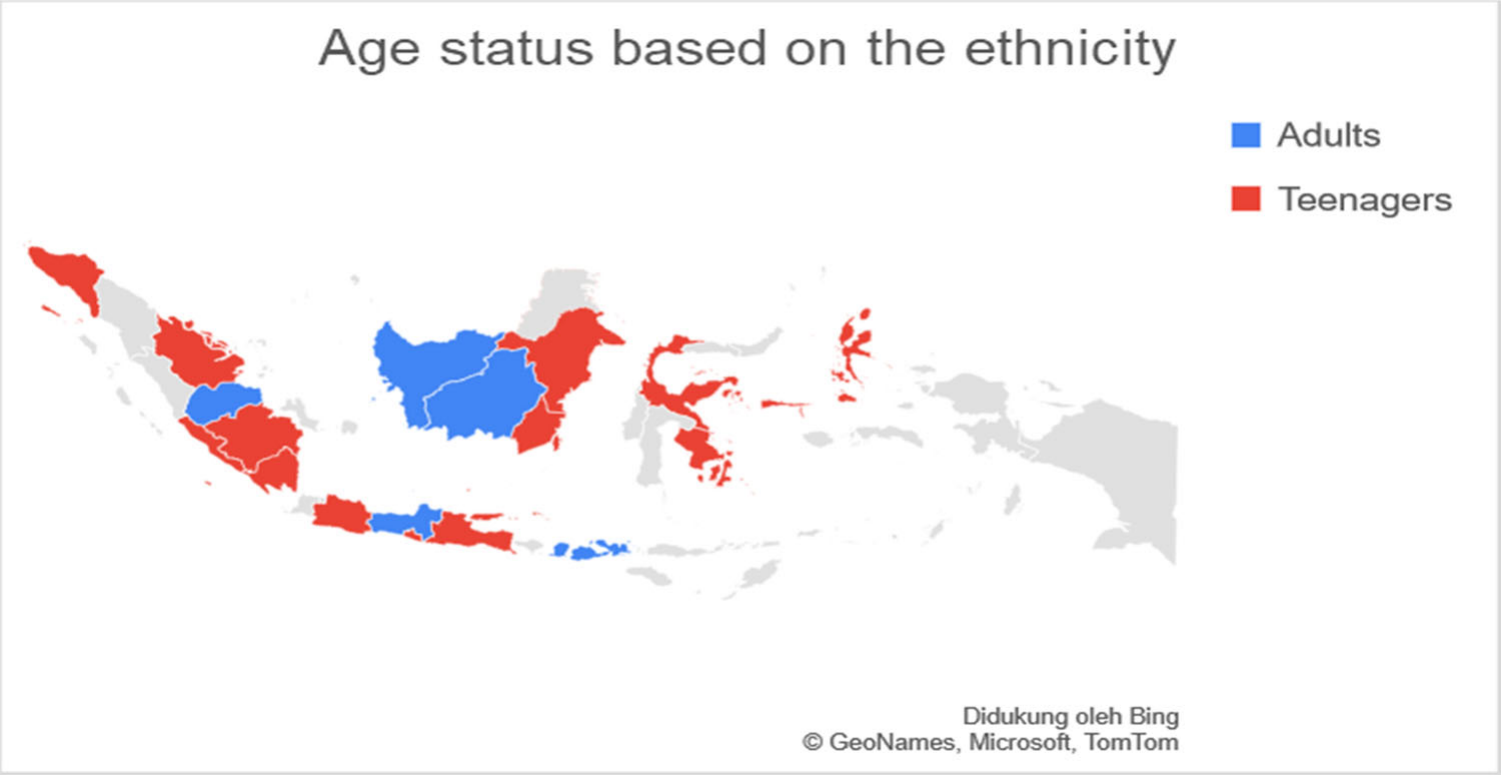
The age status is based on the ethnicity (primary source).

Based on data related to the residential of the respondents categorized into regions on Java island and outside Java island, it was found that the number of respondents in Java was more than 77.3% (*n* = 140) experiencing anxiety when compared to outside Java, which was 22.7% (*n* = 41). Respondents living on the island of Java were found to experience more anxiety with a total of 74.7% (*n* = 65) when compared with anxiety in residents living outside Java, which was 25.3% (*n* = 19.7). The residential factor in this study did not show a very strong relationship, which was obtained for a *p*‐value <0.5 with an OR value of 1.336. Figures 2, 3, 4, 5 depict the characteristics of the respondents based on their ethnicity. Figure 2 indicates that the respondents comprise students, teachers/lecturers, and educational staff, and the majority of the students' residential is widely spread in six out of seven major islands such as Sumatera, Java, Kalimantan, Sulawesi, Maluku, and Bali. Meanwhile, the respondents who experience anxiety occurred in five out of seven major islands such as Sumatera, Java, Kalimantan, Sulawesi, and Maluku. Only a small part of south Sumatera, East Kalimantan, and Bali that less likely to experience anxiety (Figure 3). The female respondents lived in five out of seven major islands and the male respondents in four out of seven major islands (Figure 4). Teenagers' age respondents were spread widely in five out of seven major islands and adults respondents in four out of seven major islands (Figure 5).

The type of work factor for the respondent group who are not working or studying at school/university showed a higher number of experiencing anxiety with 76.8% (*n* = 139) compared to the working group, namely 23.2% (*n* = 42). Respondents studied in the school/university group were found to experience more anxiety, 86.2% (*n* = 75) compared with the working group, 13.8% (*n* = 12). The type of work factor seems to have a strong relationship with the incidence of anxiety where the group is still studying in the school/university or not working tends to experience more anxiety with a *p*‐value of <0.05 with an OR value of 0.341. This shows that the group who has not worked or still studying is more at risk of experiencing anxiety by 0.341 times compared to the group that is already worked.

In the multivariate analysis by using logistic regression was found that the variables of gender, age, residential, and employment altogether have a substantial relationship with the incidence of anxiety in the education environment with a *p*‐value <0.05 and an R Square value of 0.069.

## 4 DISCUSSION

The COVID‐19 pandemic has caused various impacts, especially in terms of mental health caused by different factors such as economic problems, lack of knowledge about infectious diseases, sleep disorders, the influence of social media, quarantine or isolation of patients during the pandemic, and fear of personal and family health.^11^, ^29^ This study analyzes the impact of the pandemic on public anxiety in the education environment.

To control the confounding variables, this study applied a limitation of respondents based on inclusion criteria and exclusion criteria. In addition, this study also used multivariate logistic regression analysis to control confounding factors.^20^, ^30^ In this study, the confounding factor can be controlled properly as evidenced by the obtaining of a strong R‐Square value in logistic regression analysis, which is 0.069.^20^, ^30^

In this study, data showed a significant relationship with a *p*‐value <0.00 on the type of work factor showing a strong relationship with the incidence of anxiety. Based on this study, it is stated that the population group who does not have a job is more likely to experience anxiety during the pandemic. This is understandable since during the pandemic the population can experience anxiety caused by uncertain conditions and many layoffs throughout the pandemic. The results of this study are in line with the research conducted by another researcher^34^ which states that 87.7% of students experience severe anxiety.

On the other hand, the community population and students are also affected by the pandemic, these students also experience uncertainty in their education period where almost all learning processes are carried out online, and this form of online learning is not accessible to all the students. The difficulties that arise due to the online learning process coupled with their study load during the pandemic are very burdensome for this type of educational community group or the students, where the students had the potential to experience anxiety 0.341 times higher than the working group.

On the other hand, the age factor also affects the incidence of anxiety where the age group between 17 and 23 years is more prone to experience anxiety as much as 0.486 times compared to the age group between 24 and 64 years. This is comprehensible for the reason that teenagers have shortcomings in dealing with various problems in life and health. However, the other results of research^31^ which examined the incidence of anxiety in the elderly stated that those aged 40–60 years tend to experience anxiety more often than those aged 25–40 years.

In this study, the gender factor did not show significant results but there was a tendency that the female group experienced anxiety as much as 66.7% compared with the male (33.3%). This study is in line with other research^14^, ^32^, ^33^ which also stated that female residents are more prone to anxiety. However, the results of this study are different from other studies^(7)^ said that their research in Bangladesh stated that the female group had a lower level of anxiety (33.67%) when compared to the male (66.33%).

Another factor is the residential factor, where in this study, residence between the island of Java and outside Java did not appear to have a significant relationship with the incidence of anxiety. Although this study mentioned that the incidence of anxiety was higher in residents living on the island of Java by 74.7% compared with the incidence of anxiety in residents living outside Java (25.3%). This is undoubtedly true due to the population density on the island of Java which causes the risk of being exposed to COVID‐19, causing the community to experience high levels of anxiety than outside Java with a lower population density. The results of this study are following research conducted by Islam et al.^34^ which states that residents who live in urban areas are more likely to experience anxiety (64.9%) than those who live in rural areas (35.1%). A review of the study also said that the incidence of anxiety in Asia was 32.9%.^29^

Several alternative approaches that can be implemented by the community in dealing with anxiety during the pandemic are by performing a coping mechanism for the anxiety that arises with the intention of it does not continue to become severe anxiety.^29^ The need to reduce reading various obnoxious news about COVID‐19 on social media that can increase anxiety can also be administered. This is because someone who always follows news developments on social media tends to experience high levels of anxiety.^29^ In addition, problem‐solving in the form of providing counseling and involving psychiatrists or psychologists to educate the community can help minimize anxiety in the community.^31^ In addition, universities can also provide special programs for their students to stipulate financial and psychological assistance for the students.^34^ The use of telehealth is also found to help provide counseling to overcome the psychological impact of the pandemic.^11^

Lifestyle approaches can be taken to mitigate the impact of the anxiety. Several measures can also be taken such as physical exercises, a nutritious diet, mood balancing, and proper stress treatments, controlling and or preventing diabetes and heart failure,^1^, ^35^, ^36^, ^37^ psychological condition, maintaining a relationship, and sleep quality,^36^, ^37^ and avoiding the use of tobacco and alcohol.^37^ The use of a healthy diet such as consuming more fruits and vegetables is also a good example to alleviate anxiety and stress.^1^

### 4.1 Research strengths and weaknesses

This study has advantages including analyzing data on the impact of the community due to the COVID‐19 pandemic on the incidence of anxiety comprehended from various factors where this study is relatively new. This study also complements previous research where most of the studies only examined the impact of the COVID‐19 pandemic on university students.

The drawbacks of this study are due to the time constraints of the research and the relatively small number of samples which could be improved in future studies. Thus, conducting research with larger samples and different instruments is recommended in the forthcoming studies. In addition, the mental status of the participants did not seek in this study, hence, further studies are expected to complement the survey with the mental status.

## 5 CONCLUSION

The impact of the COVID‐19 pandemic has had a lot of influence on all aspects of life, including the impact on the education environment. This study has added important data to determine the impact of COVID‐19 in the aspect of education where this study is different from previous research. Previous research only examined a small number of students.^34^ This study complements previous research by adding other samples besides students, namely all lecturers, teachers, staff and students, and students.

In this study, by using multivariate analysis, it was found that demographic factors (age, gender, residential, and employment) altogether have a very strong relationship with the incidence of anxiety in the community in the education environment with a *p*‐value <0.05 and R square 0.069. From these various demographic factors, it was found that the age and occupation factors had a significant relationship with the incidence of anxiety and supported previous research examining the incidence of anxiety in older people during the COVID‐19 pandemic.^31^

It can be concluded in this study that although the factor of gender and demographic factors have a less significant relationship to the occurrence of the anxiety with a *p*‐value >0.05, the age and employment factors were found significantly associated with the prevalence of anxiety (*p*‐value <0.05) in the educational sector during the COVID‐19 pandemic. The demographic factors altogether had a substantial correlation (*p* < 0.05) with the occurrence of anxiety in the educational environment during the COVID‐19 pandemic.

The impact of the COVID‐19 pandemic on the populations varies widely. The evidence of the population suffering from mental health disruption persists for the duration of the pandemic. This predominantly occurred due to several factors such as fear of the suffering COVID‐19 leading to the severity, un‐pleasant extended quarantine, COVID‐19 stigma, and worrying the jobs losing due to the lockdown situation. Health and education sector may also experience the impact of the pandemic on the un‐secure feelings and un‐certainty conditions of the pandemic. Several programs, however, can be developed to diminish the impact of the pandemic on mental health.

The results of this study can be a part of recommendations for the management of anxiety during the COVID‐19 pandemic. The implications for the educational setting would be that such measures can be taken from the fact that several factors may contribute to the severity of the symptoms such as those who are female, unemployed, live in the density population, young age to identify the susceptible society. The educational sector could provide supporting programs such as mental counseling, happiness program, exercise, or yoga to reduce anxiety. In addition, such a supporting program for the opportunity for part‐time job and support may help their financial security and wellbeing. The local authority may put some strategies to enhance well‐being among those living in the local density population such as social support, and program development to eradicate poverty.

## TRANSPARENCY STATEMENT

The lead author (N Juni Triastuti) affirms that this manuscript is an honest, accurate, and transparent account of the study being reported; that no important aspects of the study have been omitted; and that any discrepancies from the study as planned (and, if relevant, registered) have been explained.

## AUTHOR CONTRIBUTIONS

**N. Juni Triastuti**: Conceptualization, data curation, formal analysis, funding acquisition, investigation, methodology, project administration, resources, software, validation, visualization, writing—original draft, writing—review & editing. **Erna Herawati**: Conceptualization, data curation, investigation, methodology, project administration, resources, software, validation, visualization, writing—review & editing.

## CONFLICT OF INTEREST

The authors declare no conflict of interest.

## ETHICS STATEMENT

The study protocol has been approved by the institutional ethics committee board Faculty of Medicine Universitas Muhammadiyah Surakarta (Approval number: 3390/B.2/KEPK‐FKUMS/III/2021). All authors have read and approved the final version of the manuscript [CORRESPONDING AUTHOR or MANUSCRIPT GUARANTOR] had full access to all of the data in this study and takes complete responsibility for the integrity of the data and the accuracy of the data analysis.

## Data Availability

The authors confirm that the data supporting the findings of this study are available within the article [and/or] its supplementary materials.
